# Protocol for tissue clearing and 3D analysis of dopamine neurons in the developing mouse midbrain

**DOI:** 10.1016/j.xpro.2021.100669

**Published:** 2021-07-22

**Authors:** Youri Adolfs, Divya D.A. Raj, Sara Brignani, R. Jeroen Pasterkamp

**Affiliations:** 1 Department of Translational Neuroscience, UMC Utrecht Brain Center, University Medical Center Utrecht, Utrecht University, Universiteitsweg 100, 3584 CG Utrecht, the Netherlands

**Keywords:** Microscopy, Model Organisms, Molecular Biology, Antibody, Neuroscience

## Abstract

Advances in tissue clearing enable analysis of complex migratory patterns of developing neurons in whole intact tissue. Here, we implemented a modified version of 3DISCO to study migration of midbrain dopamine (DA) neurons. We provide a detailed protocol starting from whole-brain immunostaining, tissue clearing, and ultramicroscopic imaging to post-acquisition quantification and analysis. This protocol enables precise quantification of DA neuron migration but can also be applied more generally for analyzing neuron migration throughout the nervous system.

For complete details on the use and execution of this protocol, please refer to Brignani et al. (2020).

## Highlights

• 3D imaging of cleared brains enables analysis of complex neuronal migratory patterns
• 3D visualization of dopamine neuron subsets using clearing and lightsheet imaging
• Detailed procedure for the analysis of neuronal subset positioning in the DA system
• Detailed methods to analyze neuronal migratory patterns during brain development

## Graphical abstract

**Figure:**
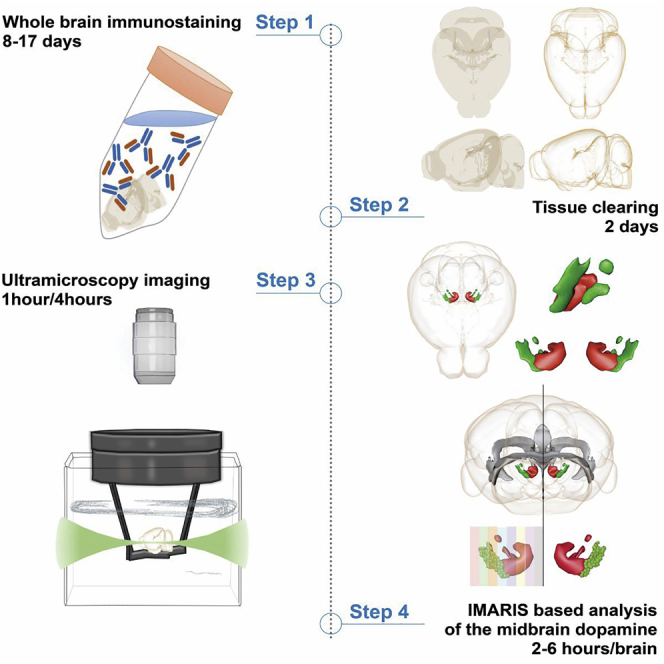

## Before you begin

### Preparation of PBSGT buffer

**Timing: 30 min**

**Table:** Prepare the PBS-Gelatin-Triton X-100 (PBSGT) buffer:

PBSGT	Final concentration	Amount	Component function
Gelatin	0.2%	2 g	Blocking
Triton-X-100	0.5%	5 mL	Tissue penetration
Thimerosal∗	0.01%	0,1 g	Sample preservation
In 1**×** PBS (pH 7.4)	1**×**	Fill up till 1L	Isotonicity, buffering

Dissolve the gelatin in the buffer by incubation at 37°C and regular shaking until it is completely dissolved.

**Figure 1:**
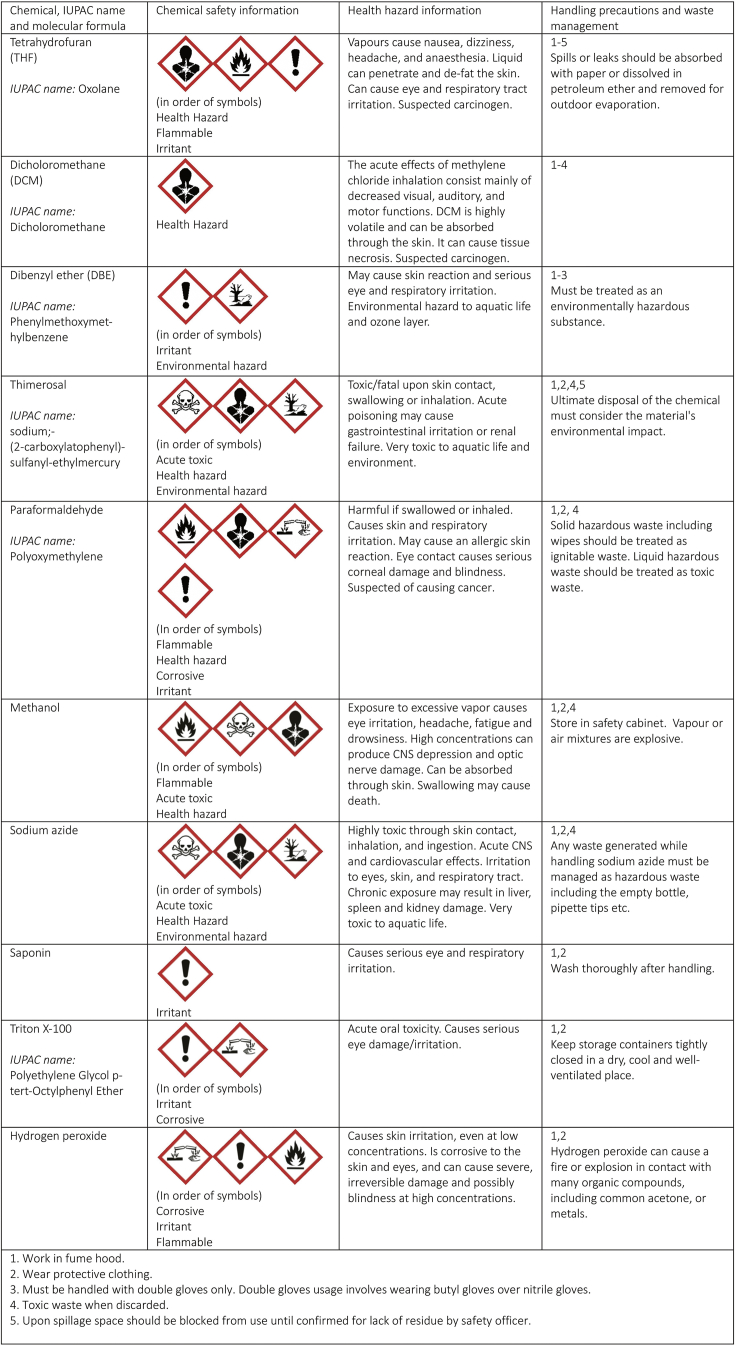
Safety information and chemical handling Information on health and environmental hazards of chemicals used in this protocol and recommended safety handling procedures and waste management.

**CRITICAL:** Thimerosal contains mercury and is acutely toxic. ∗Alternatively, sodium azide (0.01%) can be used. Sodium azide is also acutely toxic to the central nervous system (CNS) and liver. These compounds must be handled with caution in the fume hood. Safety information and waste management of both chemicals are detailed in Figure 1.

***Note:*** Once prepared, solutions containing Thimerosal/Sodium azide can be used for up to 2 weeks (when stored at 4°C).

## Key resources table

**Table:** 

REAGENT or RESOURCE	SOURCE	IDENTIFIER
**Antibodies**		
Sheep anti-TH, dilution usage (1:500)	Pel-Freez Biologicals	Cat# P60101-150, RRID:AB_461070
Rabbit anti-GFP, dilution usage (1:1000)	Life Technologies	Cat# A11122, RRID:AB_221569
Donkey anti-rabbit, Alexa Fluor 647, dilution usage (1:750)	Life Technologies	Cat# A-31573, RRID: AB_2536183
Donkey anti-sheep, Alexa Fluor 568, dilution usage (1:750)	Life Technologies	Cat# A-21099, RRID:AB_2535753

**Chemicals, peptides, and recombinant proteins**		

Methanol (analytical grade EMSURE® ACS)	Merck KGaA, Germany	1.06009.2500
Dichloromethane (DCM) (99.8% purity), contains 40–150 ppm amylene as stabilizer	Sigma-Aldrich	270997 (500 mL)
Dibenzyl ether (DBE) (98% purity)	Sigma-Aldrich	108014 (3 Kg)
Tetrahydrofuran (THF) (99.9% purity), contains 250 ppm BHT as stabilizer	Sigma-Aldrich	186562 (1 L)
Triton-X-100	Roche Diagnostics GmbH	40139421
Thimerosal	GERBU Biotechnik GmbH	1031.0010 (10 mg)
Gelatin	VWR Chemicals	24360.233
Phosphate-buffered saline (PBS) (10**×**) 1**×** diluted in Milli-Q® ultrapure water	Gibco, Life Technologies Europe B.V.	14200-067
Paraformaldehyde (PFA)	Merck KGaA, Germany	1.04005.1000
Saponin (from quillaja bark)	Sigma-Aldrich	47036 (25 g)
Hydrogen peroxide 30% (Perhydrol®- for analysis EMSURE® ISO)	Merck	1.07209.0250

**Other**		

Eppendorf Tubes® 5.0 mL, 5.0 mL, Eppendorf Quality™, amber (light protection), 200 tubes (2 bags × 100 tubes)	Eppendorf	0030119452
Eppendorf Tubes® 5.0 mL, 5.0 mL, Eppendorf Quality™, colorless, 200 tubes (2 bags × 100 tubes)	Eppendorf	0030119401
Corning® syringe filters (pore size 0.2 μm)	Merck	CLS431219
15 mL Sterile Centrifuge Tubes, Brown Color (Light Protection)	Greiner Bio-One B.V.	188283
Corning® 15 mL centrifuge tubes	Merck	430791
2 mL Microtube, wide hinge cap (pp)	SARSEDT	72691
Best® Butyl II Gloves (874)	Merck	size M: Z405299 size L: Z405302 size XL: Z402362
Nitrile gloves	Halyard	Size S: 52001M Size M: 52002M Size L: 52003M Size XL: 52004M
Agarose	Merck	11388991001
Loctite 3 g Super Glue	Loctite	212111
Moria perforated spoon (20-mm diameter)	Fine Science Tools	10370-17
Forceps	Fine Science Tools	11080-02
DURAN® graduated amber laboratory bottle, 250 mL (with cap)	Merck	218063656
Rotilabo® storage vials	Carl Roth	XC40.1
Caps for storage vials	Carl Roth	XC44.1
Quantofix Peroxide Test Strips	Sigma-Aldrich	37206
SB3 rotator	Stuart	N/A
Incu-Shaker Mini	Benchmark Scientific	N/A
Fume Adsorber TAZ19	Medite	N/A
Ultramicroscope II	LaVision BioTec GmBH	N/A

**Experimental models: organisms/strains**		

Mouse: *ACTB-Flpe:Pitx3-ITC*	Generated in-house	Brignani et al., 2020

**Software and algorithms**		

Imaris™, version 8.4–9.4	Bitplane	RRID:SCR_007370, https://Imaris.oxinst.com/
Imaris^TM^ File Converter 9.1–9.4	Bitplane	N/A https://Imaris.oxinst.com/
Imspector, version 5.0285.0	LaVision BioTec	RRID:SCR_015249, http://www.abberior-instruments.com/products/expert-line/imspector-software/
Microsoft Excel	Microsoft	RRID:SCR_016137, https://www.microsoft.com/en-gb/
Prism, version 7.05	GraphPad Software	RRID:SCR_002798, https://www.graphpad.com/

**CRITICAL:** Several chemicals and solvents used in the procedure pose severe health and environmental hazards, as detailed in Figure 1. Follow safety handling procedures and recommended waste management steps throughout the procedure in accordance to institutional guidelines.

***Note:*** The solvents used in the procedure should be of high-grade purity as mentioned in the resource table.

## Materials and equipment

• Light sheet microscopee Ultramicroscope II (LaVision Biotec GmBH) with the following specifications:
  − Olympus binocular body (MVX10)
  − Sheet optics of the instrument allow for uni/bi directional illumination, dynamic focus positioning, with thickness of 4–24 μm with numerical aperture of 0.0148–0.148
  − Olympus MVPLAPO 2**×** objective
  − Emission filters:
    • ET525/50 m (Chroma, USA)
    • ET615/40 m (Chroma, USA)
    • 676/29 (Semrock, USA)
    • 716/40 (Semrock, USA)
    • ET775/50**×** (Chroma, USA)
  − OBIS lasers (Coherent, USA):
    • 488 nm: OBIS 488-50 LX Laser (50 mW)
    • 561 nm: OBIS 561-100 LS Laser (100 mW)
    • 647 nm: OBIS 647-120 LX Laser (120 mW)
    • 730 nm: OBIS 730-30 LX Laser (30 mW)
  − Black delrin plastic lens dipping cap: Standard (with working distance 10 mm) and correction dipping cap (with working distance 5.7 mm) (Cat# LV OM DCC20, LaVision Biotec)
  − Quartz imaging reservoir (LaVision Biotec)
  − Neo 5.5 sCMOS (Andor, UK)
• Work stage for sample preparation - Medite fume adsorber, TAZ19 (Medite, Germany)
• Horizontal orbital shaker - Benchmark Incu-shaker mini (Benchmark Scientific Inc, USA)
• Rotator - Stuart SB3 rotator (Stuart, UK)

### Mouse line

Different subsets of dopaminergic (DA) neurons have distinct locations in the midbrain and specific axonal projection patterns. Based on differential gene expression profiles it is possible to genetically label different subsets of DA neurons (Poulin et al., 2016). In the ventral midbrain of *ACTB-Flpe:Pitx3-ITC* mice, DA neurons of the substantia nigra compacta (SN) are specifically labeled and can be distinguished from for example DA neurons of the ventral tegmental area (VTA) (Brignani et al., 2020). In this paper, we detail methodology that has been modified from Belle et al*.* (2014) to assess the migration of genetically labeled SN DA neurons in *ACTB-Flpe:Pitx3-ITC* mice during development. However, the described methods can be applied more generally to analyze the distribution, morphology, migration and connectivity of other (genetically or immunolabeled) subsets of DA, or non-DA, neurons.

**CRITICAL:** All animal experiments must be handled in accordance to relevant governmental and institutional guidelines. All experiments were approved by the Animal Ethics Committee of Utrecht University (Dierexperimenten Ethische Commissie) (CCD license: AVD115002016532) and conducted in agreement with Dutch laws (Wet op de Dierproeven, 1996; revised 2014) and European regulations (Guideline 86/609/EEC; Directive 2010/63/EU).

### Data management

Ultramicroscopy-based acquisition of 3DISCO samples generates large volume datasets. For example, an E13 brain scan for 2 channels set at a step size of 2.5 μm produces approximately 10 GB data with about 1000 optical images (16-bit images with 5.5-megapixel sCMOS camera). Non-tiled scans of the adult mouse brain consist of about 2000 optical images and are around 20 GB in size for each channel. The computing power required is correlated with the size of the dataset collected. For analyzing these datasets, we used a Dell workstation running Windows 7, equipped with 128 GB of RAM, a dual processor, in total 24 cores and a 8GB Nvidia Quadro K5200 video card.

Data handling follows some protocols to prevent loss of precious data. Data during scanning is always collected on a local drive to prevent loss of data due to network failure (such as during a power shortage). The raw data is saved on a backup archive server for safety. We use a fast network (10 GB) to transfer data to the analysis stations and a slower network for bulk storage (archive) of raw data. A database of sample details and scan details with file name is maintained on a project-to-project basis.

## Step-by-step method details

### Embryo collection and brain isolation

**Timing: 30 min–2 h**

Isolation and fixation of embryonic (E12-E19)/postnatal (P0-P10) *ACTB-Flpe:Pitx3-ITC* brains. For adult brain, we recommend use of the iDISCO protocol with perfusion (Renier et al., 2016; https://idisco.info/idisco-protocol/) instead of the 3DISCO protocol.

1. Brains are isolated in 1**×** PBS using a dissection microscope.
2. After brain isolation, the meninges must be removed to allow better antibody penetration.
3. Brains or whole embryos are fixed in 4% PFA in PBS (pH 7.4) without rotation at 4°C overnight (approx. 16 h).
4. The next day, PFA is removed and 1**×** PBS is added.

**CRITICAL:** Do not fix brain tissue longer than recommended. Immunostaining can otherwise be compromised due to excessive cross-linking of proteins.

***Note:*** For the analysis of embryos up to E15.5, whole embryos can be processed using this protocol depending on the quality of the primary antibody used. The anti-GFP antibody used here (Invitrogen) does not work effectively in whole embryos or in isolated brains with meninges. Therefore, when GFP immunostaining is required isolated brains without meninges are used for experiments, even for embryonic tissues (E13.5). For the analysis of E16.5 and older samples, brains are isolated from the embryo or pup.

**Pause point:** Brains can be stored at 4°C in 1**×** PBS for one week. For longer storage, add 0.01 % Thimerosal to the PBS.

### Bleaching (for whole embryos)

**Timing: 2 days**

Bleaching facilitates background reduction. This is accomplished by controlling autofluorescence derived from biomolecules such as heme, nicotinamides (NADP), and retinols (Croce and Bottiroli, 2014).

***Note:*** This step applies only to whole embryos (embryonic stages <E16.5). When isolated brains are used, skip this step and proceed to the immunostaining step.

5. Dehydration
  a. Leave the whole embryo in 1**×** PBS for 1.5 h (or longer) at room temperature (RT, 15°C–25°C) on a rotator.
  b. Remove PBS and add 50% methanol (diluted in PBS). Leave the sample for 1.5 h (or longer) at RT on a rotator.
  c. Remove 50% methanol and add 80% methanol (diluted in PBS). Leave the sample for 1.5 h (or longer) at RT on a rotator.
  d. Remove 80% methanol and add 100% methanol. Leave the sample for 1.5 h (or longer) at RT on a rotator.
6. Bleaching
  a. Move the embryo into the bleaching solution. Bleaching solution (100 mL) is prepared as 90 mL absolute methanol + 10 mL of 30% H_2_O_2_ stock (Final concentration of H_2_O_2_ in solution is 3%) and leave the sample overnight (approx. 16 h) at 4°C.

**CRITICAL:** (step 6) Do not rotate or shake the sample as this can lead to formation of bubbles which is not desirable at this step.

7. Rehydration
  a. Move the embryo to 100% methanol and incubate at RT on a rotator for at least 1 h.
  b. Substitute the solution with new 100% methanol solution and leave it at RT on a rotator for at least 1 h.
  c. Substitute the solution with 80% methanol (in PBS) and leave the sample at RT on a rotator for at least 1 h.
  d. Substitute the solution with 50% methanol (in PBS) and leave the sample at RT on a rotator for at least 1 h.
  e. Remove the methanol and add 1**×** PBS. Leave the sample at RT on a rotator for at least 1 h.

**CRITICAL:** Methanol is toxic for the CNS and liver. It is volatile and inflammable. It must be used with caution in a fume hood.

**Pause point:** Brains can be stored at 4°C in 1**×** PBS for one week. For longer storage, add 0.01 % Thimerosal to the PBS.

### Whole mount immunostaining

**Timing: variable – from 6 to 15 days**

Brains are immunostained to detect tyrosine hydroxylase (TH) and Citrine which in *ACTB-Flpe:Pitx3-ITC* label the entire DA system and a subtype of SN DA neurons, respectively.

8. Blocking
  a. The sample is incubated in the blocking solution PBSGT at RT on a horizontal shaker (70 rpm in specified shaker). For incubation timing, follow the instructions in Table 1.

**Table 1:** Antibody incubation times

*Age*	*Blocking*	*Primary antibody incubation*	*Secondary antibody incubation*	*Incubation volume*
E12	3 h	3 days	2 days	2 mL in Eppendorf
E13-18	24 h	7 days	2 days	2 mL in Eppendorf
P0	36 h	10 days	4 days	2 mL in Eppendorf

**Pause point:** Longer incubation times at this step do not affect staining outcome (We recommend a maximum of 2 days).

9. Primary antibody incubation
  a. The blocking solution is discarded and the primary antibody solution is added [primary antibodies: sheep anti-TH (1:500) and rabbit anti-GFP (1:1000) are diluted in PBSGT + 0.1% saponin]. The sample is incubated on a horizontal shaker at 37°C (70 rpm in specified shaker) as specified in Table 1.

***Note:*** Saponin removes membrane cholesterol, leaving pores in the membrane that aid tissue penetration. Saponin also facilitates the entry of antibodies into cells by forming saponin/cholesterol micelles (Seeman et al., 1973; Lucy and Glauert, 1964).

10. Washes
  a. The sample is transferred to a 15 mL Falcon tube with 15 mL PBSGT. The tube must be rotated at RT for 1 h. 6 consecutive washes (of at least 1 h each) are required.

**Pause point:** The sample can be stored in PBS at 4°C. (We recommend a maximum of 2 days).

11. Secondary antibody incubation
  a. The selection of secondary antibodies is crucial. See Table 2 for suggestions.

**Table 2:** Considerations for the choice of secondary antibodies

*Laser wavelength*	*Suggested fluorophore*	*Advantages*	*Disadvantages*
488 nm	Alexa Fluor 488, Cy2	Compatible with the 561 and 647 nm laser.	Highest autofluorescence. Least preferred channel for imaging.
561 nm	Alexa Fluor 555, Alexa Fluor 568, Cy3.5	Strong laser power; Compatible with 488 and 647 nm lasers.	Reasonable amount of autofluorescence.
647 nm	Alexa Fluor 647, Cy5	Very low autofluorescence; strong light penetration; strong laser power; Compatible with 561 and 488 nm lasers.	Most recommended channel for imaging.
730 nm	Alexa Fluor 750, Alexa Fluor 752	Almost no autofluorescence; strong light penetration.	Weak laser power^a^

a The power of the individual lasers may be different in other setups.
  b. The sample is incubated in secondary antibody solution [donkey anti-sheep 568 (1:750) and donkey anti-rabbit 647 (1:750) are diluted in PBSGT + 0.1% saponin; the solution is filtered with a 0.20 μm filter before use]. The sample is incubated on a horizontal shaker at 37°C according to Table 1. Protect from light.

***Note:*** incubations are normally performed at 37°C to promote antibody penetration. In case the antibody is not compatible with incubation at 37°C, lower temperatures can be considered.

***Note:*** The Ultramicroscope set-up comes with two options: a laser beam combiner in which multiple laser lines are arranged in one set-up or a white light laser covering a range of wavelengths (460–800 nm). Depending on the Ultramicroscope set-up used, the 730 nm laser can be positioned in a separate beam combiner which might cause minor alignment variations in the light sheet angles. In such cases, pixel-based co-localization analysis should not be performed.

12. Washes
  a. The sample is transferred to a 15 mL dark Falcon tube with 15 mL PBSGT. The tube must be rotated at RT for 1 h. 6 consecutive washes (of at least 1 h each) are required.

**Pause point:** The sample can be stored in PBS at 4°C. (We recommend a maximum of 2 days).

***Note:*** For primary and secondary antibody incubation, 2 ml Eppendorf tubes with 2 ml antibody solutions are used. Secondary antibody incubation is performed in the dark.

***Note:*** Using this protocol it is also possible to use conjugated primary antibodies or nanobodies. In this case steps 11 and 12 can be skipped.

### Tissue clearing

**Timing: 2 days**

Tissue clearing renders brain tissue transparent thereby facilitating imaging of and through whole tissue.

**CRITICAL:** Several chemicals used in the tissue clearing step are hazardous. Appropriate precautions should be applied in handling and disposing of these chemicals (summarized in Figure 1). All clearing steps must be performed in the fume hood and while using protection.

**CRITICAL:** All incubations are performed at RT in Falcon tubes wrapped with aluminum foil / dark falcons to protect the sample from light. The sample is placed on a rotator (Stuart rotator SB3, rotor diameter 27 cm, 14 rpm).

***Note:*** In the incubation steps, 100% THF solution is refreshed once. The solution for the first incubation cycle can be reused for up to two weeks, while that of the second incubation cycle is always used fresh.

***Note:*** Before every step, remove excess fluid with a tissue. Dispose tissue in hazardous waste. Fill up tubes for dehydration and clearing as much as possible without airgap to prevent oxidation of solvents.

13. Dehydration
  a. The sample is sequentially incubated in 50%, 80%, 100% and 100% solutions of tetrahydrofuran (THF) diluted in *Milli-Q*® ultrapure water at RT (Table 3). Leave the sample in 50% THF overnight (approx. 16 h) followed by incubation in 80%, 100% and 100% THF solutions for at least 1 h. Longer incubations do not necessarily improve the clearing process.

**Table 3:** Incubation times for solvents according to developmental stage

Solvent	E11.5 mouse embryo	E11.5-P0 mouse embryo	P2-P15 mouse
THF 50%	1 night	1 night	1 night
THF 80%	30 min	1 h	90 min
THF 100% (first cycle)	30 min	1 h	90 min
THF 100% (second cycle)	30 min	1 h	90 min
DCM	10 min (or until sample sinks)	20 min (or until sample sinks)	40 min (or until sample sinks)
DBE	20 min	overnight	overnight

**CRITICAL:** THF is an organic solvent that forms peroxides upon long term storage increasing the risk of explosion. This process can be suppressed by adding butylated hydroxytoluene (BHT) as a stabilizer. BHT removes the free radicals required for peroxide formation. The solvent quoted in the resource table contains BHT stabilizer. Old stock solutions should be avoided (stocks should not be used for more than 2 weeks) or checked meticulously for peroxide levels using peroxide strips.

14. Removal of lipids
  a. The sample is transferred into 100% Dichloromethane (DCM) for 20 min or until it sinks at RT.

***Note:*** THF-dehydrated tissue floats when it is placed in DCM. At the end of this step, the sample must sink. If the sample did not sink, lengthen the DCM step (step14). If the sample does not sink after 4 hours, it probably contains an air bubble in which case it is safe to proceed to the next step.

15. Clearing
  a. Move the sample to 100% Dibenzyl ether (DBE) overnight (approx. 16 h) at RT.

**Figure 2:**
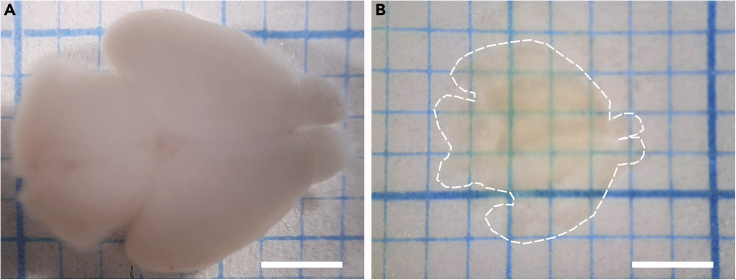
Clearing of postnatal mouse brain tissue (A and B) (A) Uncleared P0 brain sample, and (B) P0 brain sample cleared using 3DISCO. The borders of the cleared brain are indicated with a dashed line. Scale bar = 2 mm

***Note:*** After DBE incubation, the sample must be transparent, having the same refraction index as DBE (1.56) (Figure 2B).

**Pause point:** The sample can be stored in DBE at RT in brown glass vials until imaging. The sample must not be stored in the fridge, otherwise it loses its clarity and will become opaque. In our experience the samples can be stored for months without a significant decrease in staining intensity, although we had some rare examples in which this was not the case. It is therefore advisable to image your samples within a couple of weeks after finishing tissue clearing.

### Ultramicroscopy imaging

**Timing: 1 hour (E13.5 brain) / 4 hours (P0 brain)**

16. Microscope calibration: check whether the alignment of the light sheets has been recently evaluated and adjusted with the alignment tool (150-000-520). See troubleshooting section.
17. Check for sufficient clearing solution in the chamber. See troubleshooting section.

**CRITICAL:** The light sheet objective should always be handled with a dipping cap to avoid damage to the lens (unless a special DBE resistant lens is used). Handling DBE should be done with utmost care. Any spillage of the fluid onto the stepping motor located below the clearing solution chamber will cause damage.

**Figure 3:**
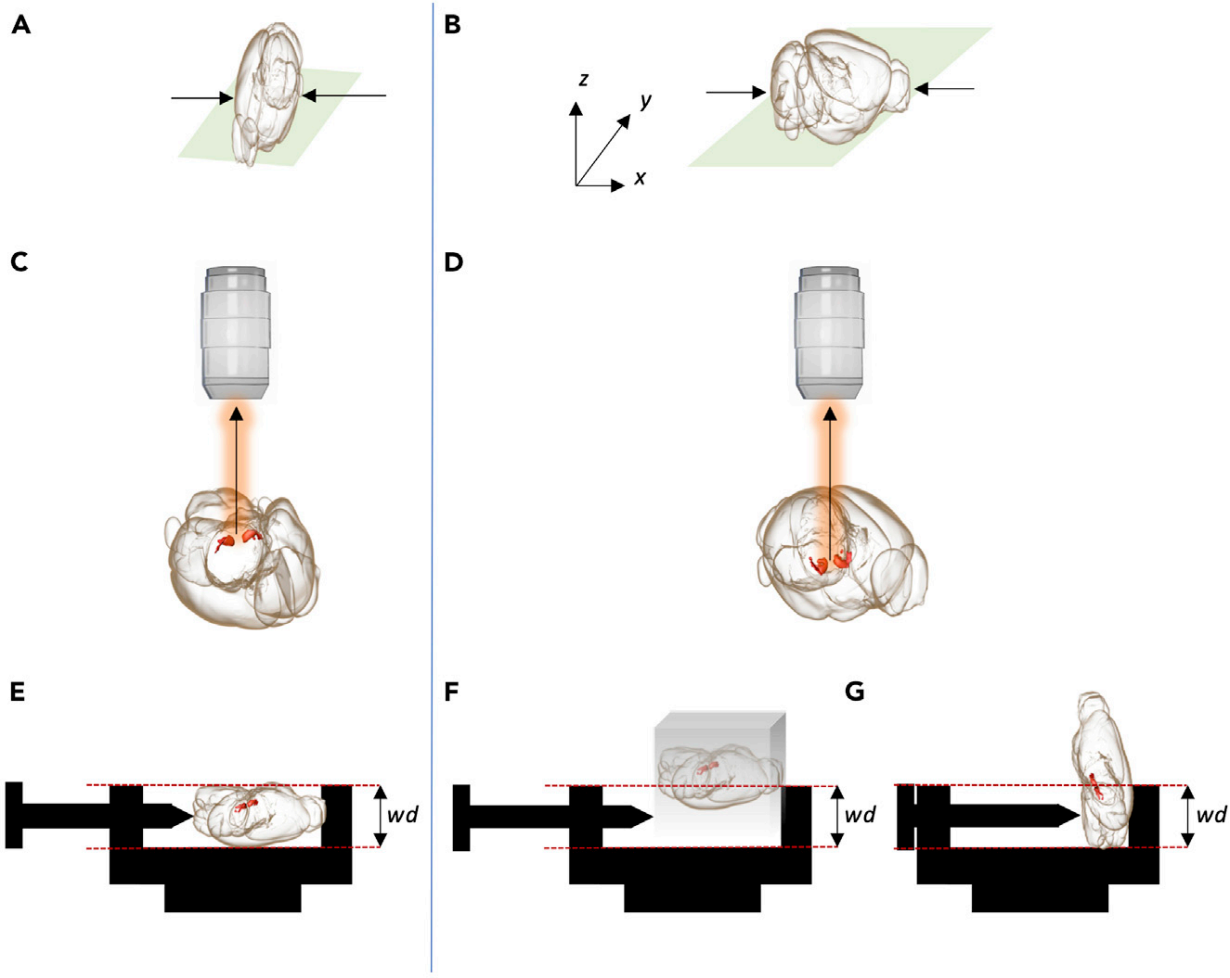
Mounting of brain samples (A and B) Mounting of samples as in A and B allows excitation light to pass through the width and length of the brain, respectively. In A the distance excitation light has to travel is shorter and therefore this way of tissue mounting is ideal for imaging. The arrows indicate the direction of the incident light. (C and D) Dopamine (DA) neurons are located in the ventral midbrain. Therefore, it could be an option to mount the brain sample such that the medial sulcus is up and the cortex down (C) which reduces the distance emitted light (arrow) has to travel to the lens. In D, emitted light has to travel a long distance in the brain tissue. (E–G) DA neurons should be present within the working distance (wd) of the objective following mounting (E). If the sample is not trimmed well, e.g., when cleared in an agarose block (F), or mounted in the incorrect manner (G), the DA system will not be positioned entirely within wd.

**Figure 4:**
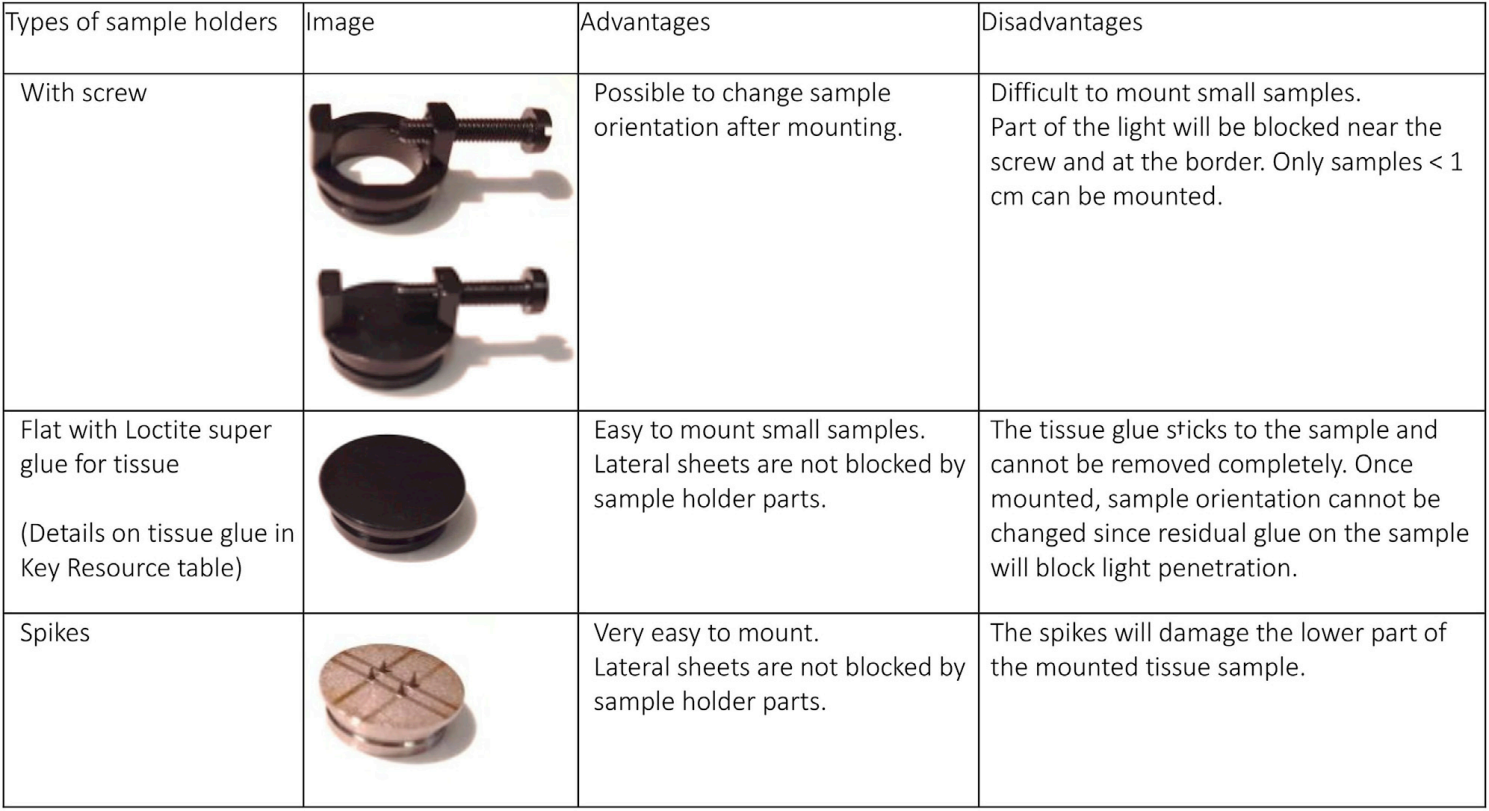
Comparison of sample holders Differences between sample holders and (dis)advantages of the different holders.

18. Deciding on optimal sample orientation and mounting method: A number of points are to be considered when deciding on sample orientation and mounting. Figure 3 summarizes a few critical points.
  a. Although the tissue sample is cleared, longer paths of the excitation light through the sample will lead to more diffraction. It is therefore important to mount the sample such that the propagation length of the excitation light is as short as possible (Figures 3A and 3B).
  b. If the region of interest (ROI) is located on one side of the sample, the sample should be mounted such that the ROI is at the top. This minimizes the depth the emitted light has to propagate through the sample to reach the objective (Figures 3C and 3D).
  c. The sample should be oriented such that imaging the full ROI does not exceed the working distance of the objective. (Figure 3E–3G).
    ***Note:*** Moving the sample too close to the objective may cause tissue damage, tissue dropping from the sample holder or damage to the dipping cap. When the step motor is moved to the lowest Z position, the sample should not extend above the sheet thereby making it difficult for the user to predict the actual working distance (Figures 3F and 3G).
  d. Sample alignment should be chosen in accordance with the alignment of the laser scan as this significantly impacts the quality of the final scan. The quality of the 3D reconstruction will be higher in the XY plane as compared to the XZ and YZ planes. The axial (Z) resolution is limited by the thickness of the sheet.
  e. Choice of sample holders (See Figure 4 for a comparison of samples holders).
    ***Note:*** There must be clear rules for which parts of the Ultramicroscope can be handled with/without gloves. For instance, users should handle sample mounting with gloves and the microscope without gloves to keep it free of clearing solution.
    ***Note:*** Small samples that are challenging to mount with screw holders or with glue can be embedded in 1% agarose to facilitate mounting. It is important to perform the embedding before tissue dehydration after the immunostaining. Agarose embedding can cause tissue damage, in our experience, embedding works well for spinal cords and organoids and less for embryos and embryonic brains. See troubleshooting section.

19. Gently but firmly mount the brain in the sample holder and lower it into the cuvette of the microscope. Pay attention to the force applied to the sample while securing it in the holder. The sample has the consistency of glass and with higher forces it can crack. On the other hand, if not firmly done, the sample runs the risk of dropping out when immersed into the clearing solution when not firmly put in the sample holder.
20. Switch-on one visible laser with low laser power (10–20%) and high exposure time. Subsequently, move the sample in the Z direction until the light sheet is on top of the sample. This is done to minimize the risk of crashing the objective into the sample while lowering the objective into the clearing solution.
21. Choose the width of the light sheet and check whether the entire sample is illuminated.

**Figure 5:**
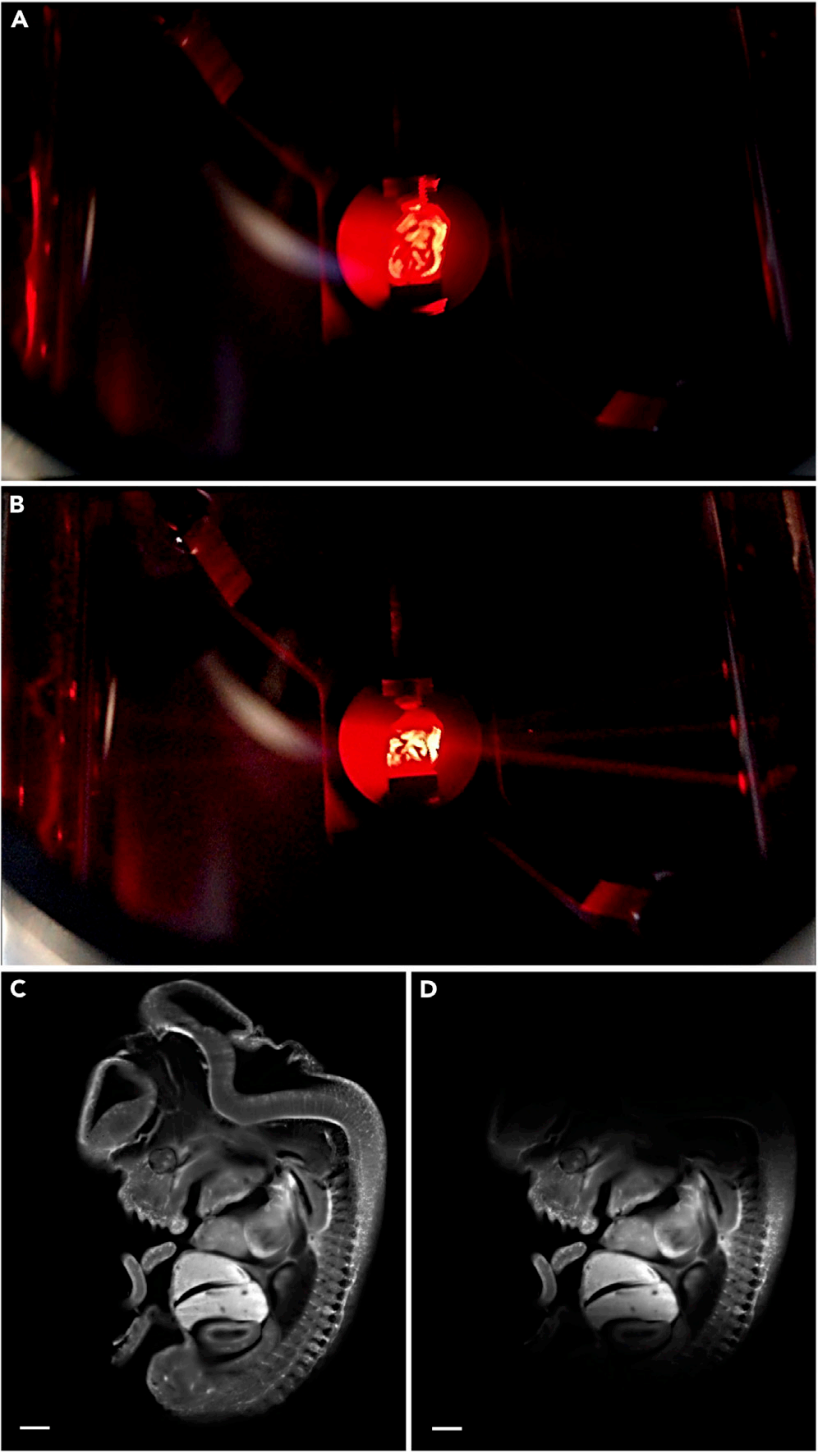
Relationship between sheet-width and sample coverage The relationship between sheet-width and sample coverage is shown here. 30% sheet width illuminates the entire sample (E13 mouse embryo) in this example showing complete sample coverage (A) whereas 10% sheet width covers only the middle aspect of the sample (B). (A and B) show sample coverage with sheet width used. (C and D) show corresponding autofluorescence images made with the Ultramicroscope II using sample settings as in A and B, respectively. Scale bar = 300 μm.

***Note:*** In this setup two types of dipping caps can be used: a standard (Figure 7A) and a correction (Figure 7B) dipping cap. With the standard dipping cap, the diaphragm should be opened half-way in order to reduce the strong spherical aberration that is caused by this type of dipping cap. The correction dipping cap incorporates a lens to correct spherical aberrations allowing the diaphragm to be fully open. The correction dipping cap has a shorter working distance (5.7mm instead of 10mm).

Figure 5 shows the relationship between sheet width and sample coverage while imaging.

***Note:*** By default, the tissue sample is illuminated by three beams: one central beam and two lateral beams projected at a small angle. The lateral beams can minimize artifacts, like horizontal dark stripes, caused for instance by air bubbles or pigmentation. The use of lateral beams, however, comes at the cost of axial (Z) resolution. This is because the waists (this is where the beam is thinnest, the Gaussian beam stops converging here and starts diverging) of the three beams can be aligned to each other only in the center of the field of view (FOV). When the three beams are combined, the sheet will only keep the same thin waist over the same length in the center of the FOV, at the top and the bottom of the FOV the thin waist will be significantly shorter (for a detailed description of this phenomenon see Ariel, 2018).

**Figure 6:**
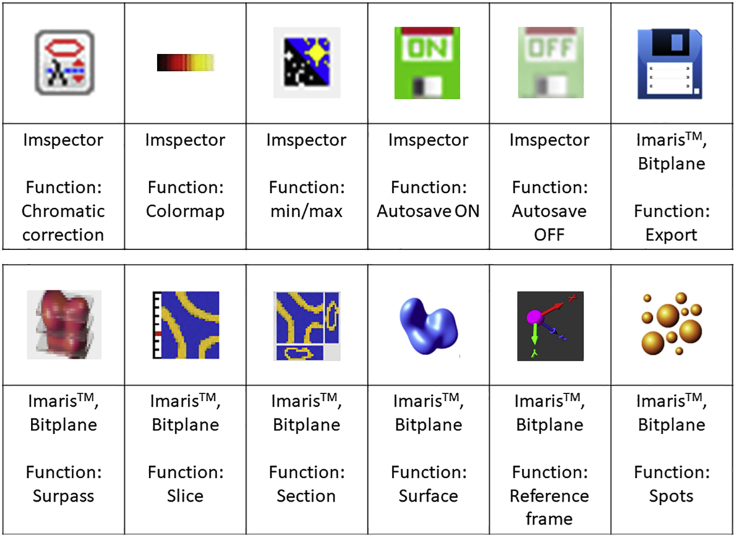
Software icons used for imaging and analysis Images of icons as they appear in the graphical user interface (GUI) of the software are shown here. The corresponding software and the function of the icon is also indicated.

***Note:*** In order to prevent excessive movement in Z while inspecting the sample, it is possible to mark the upper side of your sample as “0” using the “set as zero” button, like this it can be easily monitored, if the current position does not exceed the working distance.

22. Use the camera to focus (the microscope does not have an ocular). Once inside the clearing solution, use the min-max intensity function (accessible with F9-F10 in keyboard or via icon as shown in Figure 6) to visualize the sample and adjust the focus in small steps. The min/max function changes the scaling of the colormap such that the lowest pixel on the displayed image is set as the lower boundary and the highest pixel as the upper boundary of the color map.

23. Perform a sample inspection at low laser power to check the quality of the sample. (See troubleshooting section for possible solutions).

***Note:*** If the sample is well cleared and has small dimensions, the sample can be imaged unidirectionally. The alignment of the right and left image is never perfect, even after calibration of the microscope. For estimating if illumination from one side is ideal, the horizontal focus can be moved to the outer edge of the sample away from the illumination direction of the sheet to check if the sample is still in focus at this position. Using unidirectional illumination also significantly decreases scanning time.

***Note:*** For quantitative comparisons of different brain samples, microscope configuration and settings that may influence intensity values and imaging resolution must be applied consistently (e.g. between wild type and knockout brains). This includes settings of the diaphragm of the zoom-body, type of dipping cap, exposure time, laser power, light sheet width, light sheet NA, magnification, and step-size (See Table 4).

**Table 4:** Example of saved microscope settings on Ultramicroscope II

Parameter	Microscope settings
Sample name^a^	3D20200416-01_P0_ITC
Tissue description	Whole P0 ITC mouse brain, 3DISCO cleared. Stained for TH and citrine.
Scan date	18-May-2020
File name^b^	3D20200416-01_P0_ITC_2x
Name of microscope	Siedentopf (Right)
Sample orientation	Coronal
● Plane Z0000 contains:	Rostral side
● X0 contains	Dorsal side
● Y0 contains	Ventral side
Imspector software version	Pro 7.0.123.0
Diaphragm zoom body	Full Open
Correction collar	3.5
Magnification on Zoombody	2
Dipping cap	CDC (Correction lens)
Objective + Dipping cap	2.152
Effective magnification	4.304
Exposure time (ms)	199
Sheet NA	1.1483
Sheet width	30.00%
Stepsize (μM):	2.5
750 nm laser (33mW):	N.A.
647 nm laser (120mw)	50.00%
561 nm laser (100mw)	60.00%
488 nm laser (55mw)	N.A.
Sheet direction	Left
Dynamic focus	15

a Explanation of sample name: (name staining method (in this example 3DISCO), date in YYYY-MM-DD format (for optimal sorting files) this was the date the staining started, sample number, age, genotype).

b For the file name of the image, the sample (code) name was used, so it could be easily traced back in a digital lab journal, additionally the zoombody magnification was added to the name.

24. Set the objective correction collar to the appropriate position (3.5 for correction dipping cap and 5 for standard dipping cap).
25. Select the instrument mode which matches the objective/dipping cap and hereafter choose the appropriate magnification of the zoombody and zoom in the software. This is very important since processes like dynamic focus position and scaling are determined by both settings (See troubleshooting section for more details).
26. Select the correct mode of measurement (e.g., 3D acquisition, Multi-color 3D). For multi-color imaging, select the appropriate laser/filter combinations.
27. Determine the appropriate sheet NA. A higher NA results in a thinner light sheet but only in a small region near the light sheet focus. A lower NA results in a thicker light sheet but a more even axial (Z) resolution over the FOV. In order to get a high axial (Z) resolution with a high sheet NA over the whole FOV use dynamic horizontal focusing (See step 31).
28. Enter the step-size in the “Scan Range”. As a general rule of thumb, the value used is half the thickness of the sheet. (For example, with a sheet thickness of 5 μM, a step size of 2.5 μM was used). Choosing a too small step-size will lead to oversampling, longer imaging time and increased risk of bleaching without improving the axial (Z) resolution. A too large step-size on the other hand will cause decreased axial (Z) resolution. It is advisable to experiment with several step-sizes and check the axial resolution by inspecting the scan in XZ and YZ orientation.
29. Start with focusing on the dye with the lowest wavelength. For the best focus put the horizontal focus on a thin feature in the image (such as axons), zoom in digitally and adjust focus.
30. Adjust the focus of the other channels by adapting the chromatic correction settings (via icon shown in Figure 6).
31. Select “Dynamic focus” in order to get an even axial (Z) resolution over the whole FOV. Select the dynamic focus range and adjust it until it covers the full ROI. Based on this (and factors like magnification, instrument mode etc.) the number of horizontal focus steps necessary to achieve a homogeneous axial (Z) resolution is approximated. The more horizontal focus steps, the longer the image acquisition time. It is therefore wise to experiment with different numbers of steps before applying one to all the samples to be compared in a dataset.
32. After all the parameters are set for the experiment, set exposure time and laser power for imaging. Ideally for imaging exposure time is low (in order to decrease imaging time) and laser power just enough to image the signal without causing bleaching. Illumination intensity is not only determined by the laser power, but also by sheet width and sheet NA.
33. Choose a contrast range for your image. High contrast images are facilitated by having a broad range of grayscales in the image. How broad this range should be, depends on whether the signal-to-background ratio is high enough to depict/analyze the staining well.

**Figure 7:**
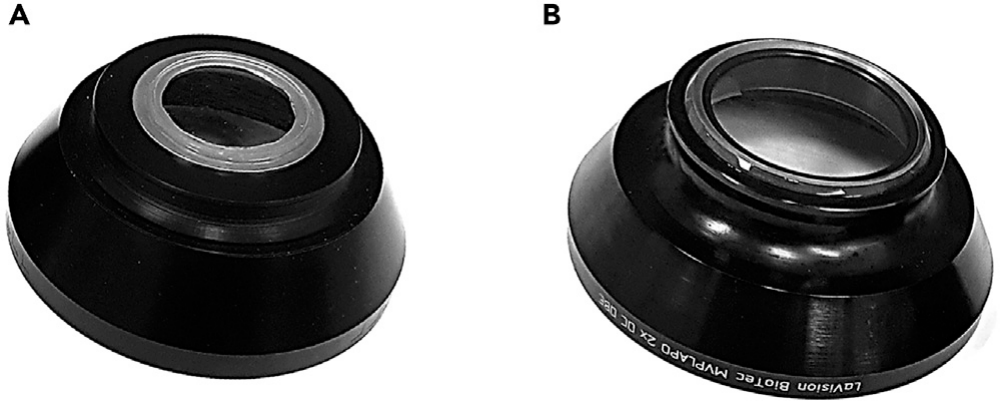
Dipping caps A standard dipping cap (A) allows light to pass through without correction for spherical aberration. A correction dipping cap contains a lens that corrects for spherical aberration when light passes through (B).

To be able to use the full range of your camera while not overexposing the sample, use the following strategy: 1) Choose a color map in the Imspector software in which the pixel with the highest intensity is depicted in another color (In the colormap shown in Figure 6 the most intense pixel is depicted in blue), 2) Now set the upper boundary of the colormap to 65534 (The Andor camera is a 16-bit camera with 2^16^ gray values. Because 0 is also counted as a gray value (black) the formula should be 2^16^-1 = 65535, but because of a software bug the highest pixel (blue pixel in our case) is not shown in that color. Therefore, subtract one extra gray value 2^16^-2 = 65534), and 3) Now change the exposure time and or laser power such that the staining is clearly depicted while there is no overexposure throughout the sample (seen by the blue pixel). Aiming for the highest contrast can come at the cost of imaging time and/or at the risk of bleaching.

34. It is highly recommended to autosave the scan (in Figure 6 it is shown as enabled or disabled) with a unique code and magnification details. The filepath (this includes folders) should be <256 characters, without spaces to avoid processing errors in image analysis software. The date and time are automatically saved in the image name.

***Note:*** In the advanced setting for autosave, select “Add meta data only to first image of series”. This will reduce the overall scan size. However, it is important to note that this first tiff image should be used to open your data with proper scaling.

### Imaris™-based analysis of DA neuron subset position and migration

**Timing: 2–6 hours/brain (Analysis time is influenced by experience)**

Using Imaris™ software, the DA system of the ventral midbrain is selectively visualized and Citrine^+^ SN neurons positioned in this area are detected. These neurons are divided in groups based on their relative position to a reference frame in the X, Y, and Z axes. The number of neurons in each group is determined. When performed at different developmental stages, this procedure allows analysis of the migration of Citrine^+^ SN neurons.

**Figure 8:**
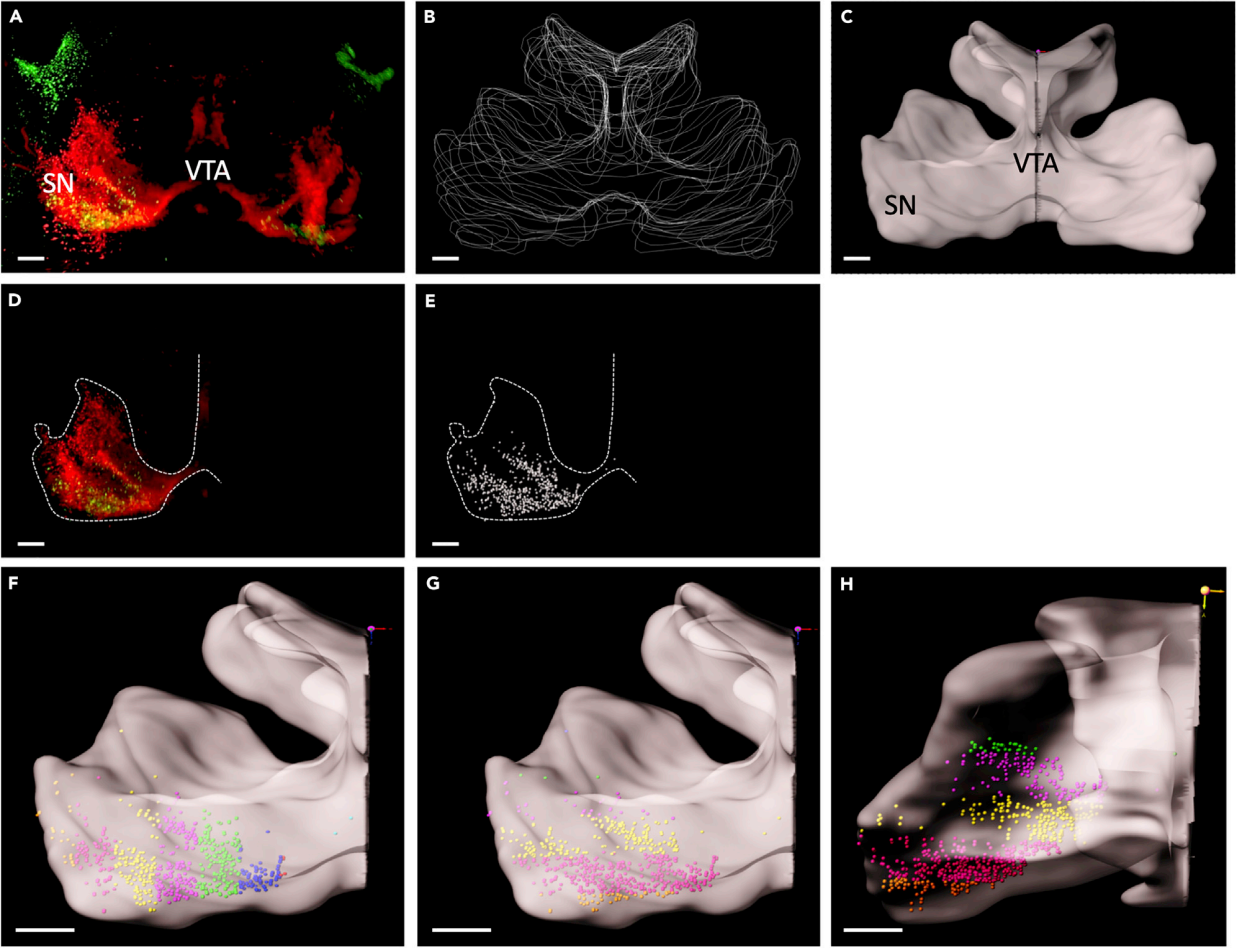
Visualizing Citrine-positive neurons within a 3D surface based on TH labeling (A) Wholemount immunostaining for tyrosine hydroxylase (TH) (in red), to label the midbrain dopamine (DA) system, and Citrine (in green), to label substantia nigra (SN) DA neurons, followed by tissue clearing and Ultramicroscope light sheet imaging (sample shown is from a P0 mouse). Dorsal is to the top. (B–E)(B) A collection of manually drawn boundaries based on the TH signal. Imaris™ uses these manual boundaries to create a 3D surface, e.g., in this case of the DA system (C). It is possible to isolate the Citrine^+^ cells (D) as data points within the TH-positive DA system (E). Bins can be created equidistant to each other from a reference frame and these can be individually analyzed in multiple directions. Each individual Citrine^+^ neuron is shown as a spot, which is displayed in a unique color for each bin. (F–H) show medio-lateral, dorso-ventral and rostro-caudally drawn bins. Scale bars,100 μm.

Below the analysis procedure is explained in detail. Snapshots of the steps involved are shown in Figure 8. The complete procedure, as described below, can also be viewed in an instructional video (Methods video S1).

Methods video S1. 3D Analysis of dopamine neurons in the developing mouse midbrain In this video the complete analysis as described in the chapter “Demonstration of Imaris™ based analysis of DA neuron subset position and migration” is shown using a whole-mount and 3DISCO cleared, TH and Citrine immunostained *ACTB-Flpe:Pitx3-ITC* P0 mouse brain imaged with a Ultramicroscope II. Related to step 36-42.

We used Imaris™ software, but technically it should be possible to perform these kinds of analysis with other software packages (e.g., Fiji or Aivia™).

**Figure 10:**
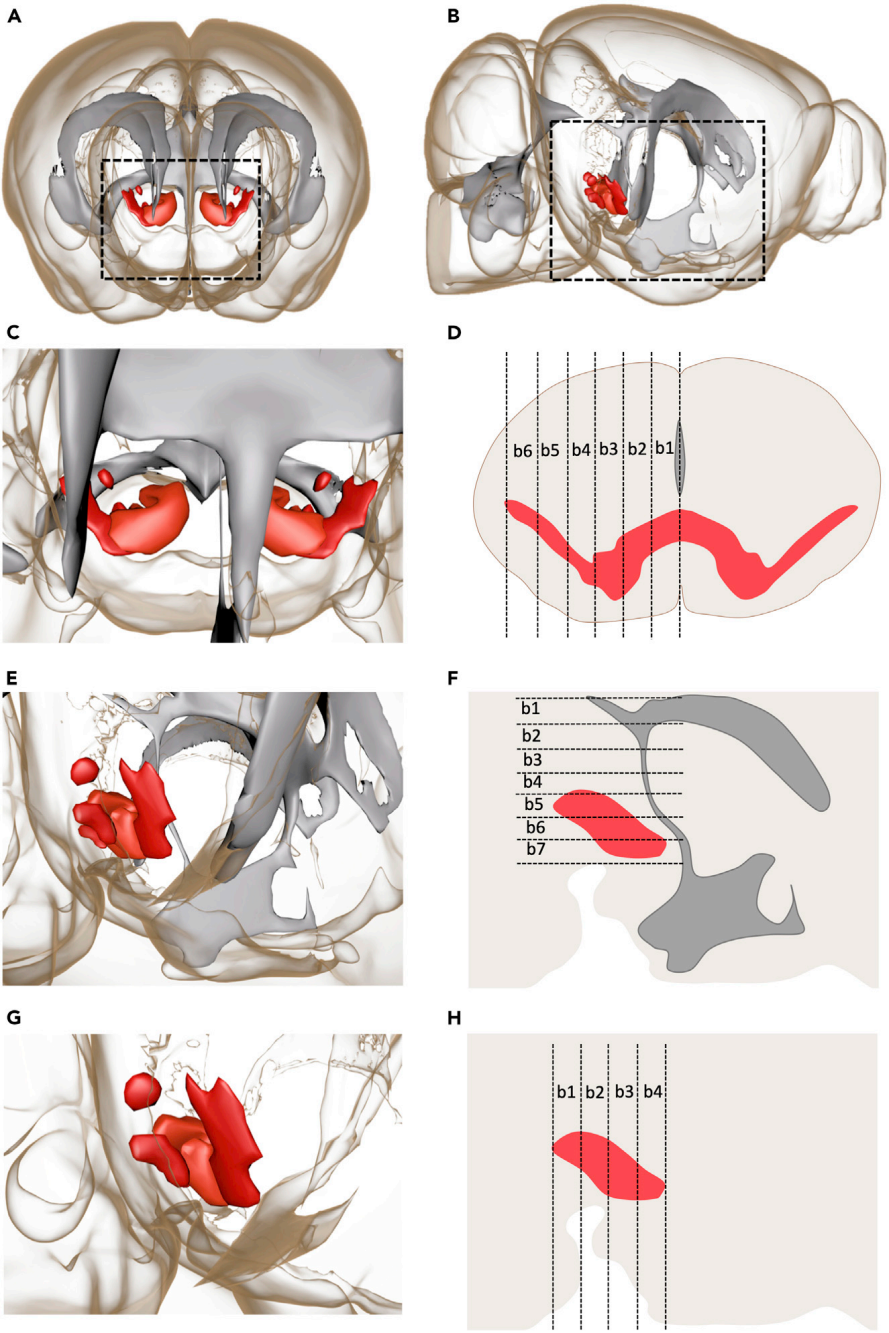
3D rendering of the dopamine (DA) system and ventricular system in the P0 brain (A and B) Front (A) and side (B) views of the P0 brain with the TH-positive DA system shown in red and the ventricular system shown in gray. The boxed area in A is shown at higher magnification in (C). The ventricular structure at the midline acts as a histological landmark for setting the central image plane from which equidistant planes are drawn until the ventral-most TH-positive point. This enables medio-lateral position analysis by creating bins (b1-6) as shown in (D). The boxed area in B is shown at a higher magnification in (E). The top edge of the ventricular structure and the upper edge of medial sulcus act as a histological landmark for setting the top and bottom image planes within which equidistant planes are drawn for creating dorso-ventral bins (b1-7) as shown in (F). For rostro-caudal analysis, the rostral-most and ventral-most TH-positive points are used to draw the boundary image planes (G and H) and between these equidistant planes are drawn (b1-4).
  c. Adding ‘Planes’, by ticking the ‘Visible’ checkbox in the ‘Reference Frame’ wizard in xy, xz and yz axes can help to visually cross-check if the orientation and rotation angle of the reference frame is set as desired.
  d. Place the reference frame at the most medial, caudal, ventral position of the surface and take care that the axes are aligned properly so they resample the medio-lateral, rostro-caudal and dorsal-ventral positions.

35. Convert file formats to facilitate the analysis.
  a. Convert acquired light sheet files (ome.tiffs) to Imaris™ file (.ims) format by using ‘Imaris™ File Converter’, by dragging and dropping the first file of the Z stack (Z0000) to the file converter. This procedure shortens analysis time in Imaris™.
  b. Store data on a local system to speed up analysis time in Imaris™.

36. Inspection of the DA system in 3D. (See Methods video S1, 0:00:06)
  a. Open the sample in the 3D view using the ‘Surpass’ function (Figure 8A).
    (Surpass>View> 3D view. Alternatively, 3D view can be accessed using Ctrl+5).
    (Icon shown in Figure 6)
  b. The sample can be inspected using a variety of functions, such as:
    i. rotation of the 3D image, positioned in the navigate function.
    ii. creation of optical slices of the 3D image using ‘Slice Viewer’ function (View> Slice or Ctrl+1).
      (Icon shown in Figure 6)
    iii. sectioning the 3D image at depth of choice (View>Section or Ctrl+2).
  c. Check for the following anomalies in the sample by visual inspection (see troubleshooting section for possible solutions):
    i. physical damage due to procedure in the ROI.
    ii. inadequate antibody penetration in deep brain regions due to antibody depletion or oversaturation.
    iii. out-of-focus imaging of cells of interest.
    iv. loss of data from one or more image planes due to sample movement during image acquisition.

37. Visualization of the DA system in 3D. (See Methods video S1, 0:01:20)
  The ‘Surfaces’ visualization creates an artificial solid object within a volume object in order to visualize/manipulate these objects in isolation. In our case, this procedure consists of manual drawing of a surface using TH immunostaining as template to isolate the DA system.
  ***Note:*** According to our experience, automatic creation of a surface does not allow a complete and specific selection of the DA system. Once the surface is created by manual drawing as explained below, a new channel is created wherein the voxels outside the DA system are set to zero and become black.
  a. Open the image in 3D view (as above) and add a ‘Surface’ (3D view> Surface).
    (Icon shown in Figure 6)
  b. In the surface wizard, select ‘Skip automatic creation, edit manually’ and deselect the ‘Volume’’ function. This enables the user to visualize the ‘Slice View’ function which is necessary for drawing the mask of the DA system.
  c. The camera type should be in the ‘Orthogonal’ mode (instead of ‘Perspective’) to keep the image in a straight position.
  d. Select the ‘Draw’ tab and, using the slice position slider, determine the position where the boundary of the area containing TH-positive cells is clearly defined.
  e. Pick the drawing mode, depending on the staining/task. In our study the ‘Click’ function selected.
  f. First adjust the mode of the pointer from ‘Navigate’ to ‘Select’ by pressing Esc and then select ‘Draw’.
    ***Note:*** It is not always necessary to draw at each slice position. It is possible to skip several planes depending on how much the boundary of the TH-positive area changes in shape among different planes. In this example, we skip between 5-20 slice positions (Figures 9A–9C).
    **Figure 9:**
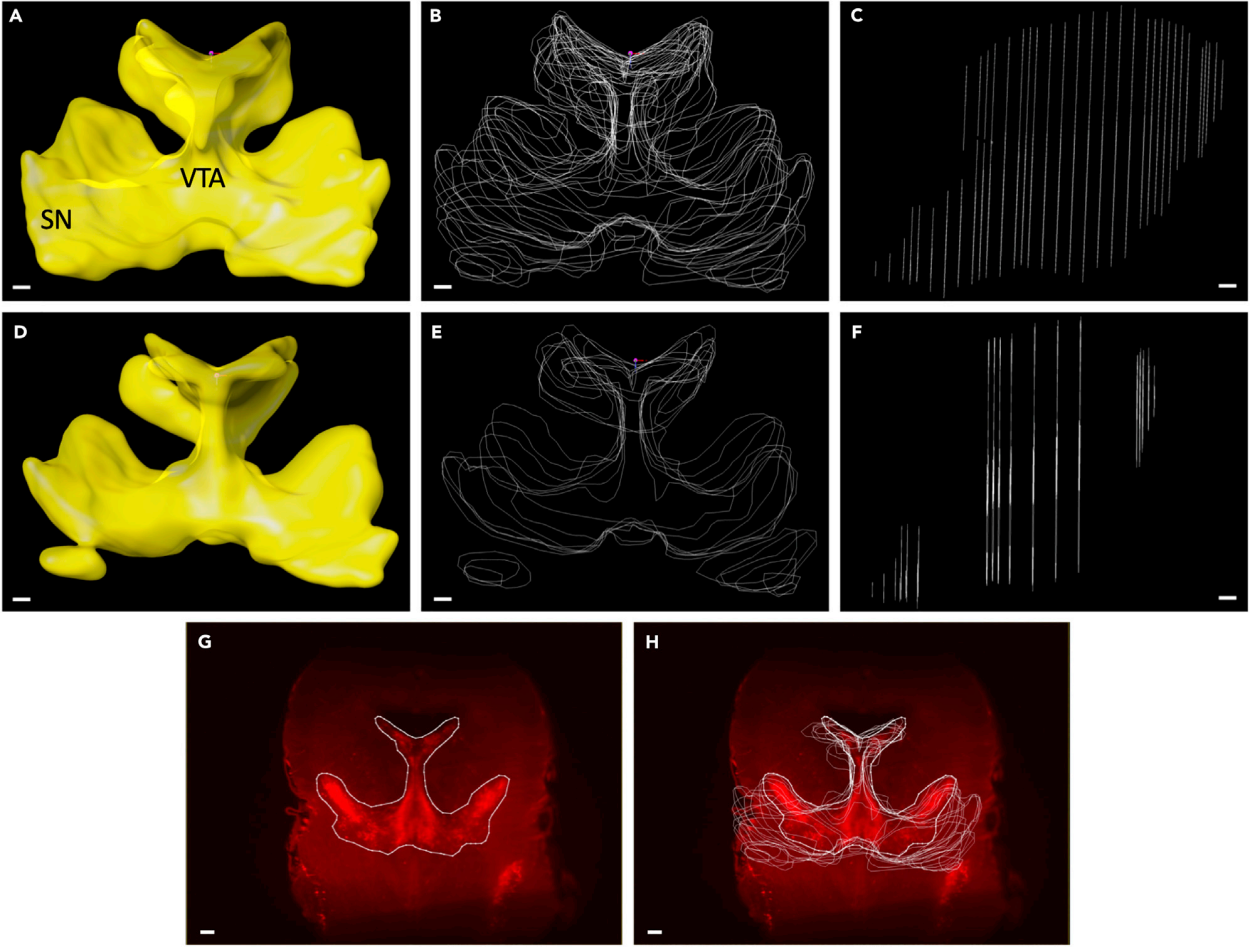
3D rendering of the TH-positive dopamine (DA) system (A–C) The generation of multiple cross-sectional drawings (B) allows 3D rendering of the entire TH-positive DA system (sample shown is of a P0 brain) (A). In C, each line represents a single cross-sectional border drawing. Sagittal view. (D–F) The use of fewer cross-sectional drawings (E and F) allows the generation of a basic structure (D), which can be fine-tuned by adding more cross-sections at levels where more details are needed, as judged by visual inspection. (G and H). Representation of TH boundaries showing only the plane being drawn (visibility “None” mode; G) or showing all planes drawn (visibility “All” mode; H) in one field of view. Scale bars = 100 μm.
  g. Draw with a left mouse click. If a point is not placed well, it can be moved by turning off ‘Draw’ and pressing ‘T+left click’. Also, additional points can be added by activating ‘Draw’ and pressing ‘T+left click’.
  h. When the first plane is drawn, move to the next slice position and repeat the same process (Figure 8B).
    ***Note:*** While drawing TH boundaries in different planes it is helpful to simultaneously visualize the drawings in other slices. This can be achieved using the ‘Contour > Board’ subtab of the ‘Draw’ tab by putting the ‘Visibility’ to ‘All’ or ‘Next’ to see all or only see the previous drawn slice(s), respectively (Figure 9H). When ‘Visibility’ is changed to ‘None’ only the drawing in the current plane is visualized (Figure 9G).
  i. When the complete ROI is drawn press ‘Create Surface’ (Resolution, shape and impact values were unchanged in our analysis) (Figure 8C).
    ***Note:*** Sometimes the quality of immunostaining in one hemisphere is low, in this case it is possible to use only one half for analysis by digitally sectioning the surface in half. To divide the surface into two halves, select the ‘Edit’ tab and put the pointer on ‘Select’ mode (Esc). Then press ‘Shift+left click’ on the position you want to split and select ‘Cut Surface’. The cutting function works only when the ‘Edit’ tab is active.

38. Setting coordinates for the DA system. (See Methods video S1, 0:04:53)
  In order to facilitate data analysis of different samples, 3D brains were oriented and aligned along a specific spatial coordinate. For this the ‘Reference frame’ function was used. Reference frames are orthogonal coordinate systems that can be positioned manually to mark coordinates in the 3D space.
  a. Select ‘Reference Frame’ (3D View> Reference Frame). The reference frame will appear in the upper left corner of the image.
    (Icon shown in Figure 6)
  b. To move the Reference frame to the correct position, select ‘Navigate’ mode (Esc)∗. Then press ‘Ctrl+right click’. In order to orient the reference frame to the desired location, rotate the reference frame by pressing ‘Ctrl+left click’ and move it with the click and drag function of the mouse.
    **CRITICAL:** It is important to choose histological landmarks that are uniform between samples in order to be able to perform this analysis. For example, for medio-lateral quantification of DA neuron migration the midline along the ventricles was chosen to set the reference frame (Figures 10C and 10D). For dorso-ventral quantification, the most upper edge of the ventricles was chosen as the histological landmark (Figures 10E and 10F). For rostro-caudal quantification, the most caudal point of the TH mask was the selected histological landmark (Figures 10G and 10H). One reference frame/sample for all axes was used throughout the analysis.
    ***Note:*** Imaging without any staining in the 488 nm channel can be used to aid the identification of the above-said histological landmarks (due to the high level of autofluorescence in this channel).

39. Isolating the DA system for analysis. (See Methods video S1, 0:05:59)
  The creation of a masked channel allows defining a region of interest (ROI) by nullifying voxels outside the mask such that they are ignored for further analysis. This allows analysis of Citrine^+^ cells only within the TH^+^ DA system, as voxels outside the TH mask are excluded from analysis.
  a. Select the (split) surface you are going to use for analysis, by placing the pointer in the ‘Select’ mode (Esc) and then left-click on that part of the surface (the selected area should turn yellow). In the surface wizard, with the ‘Edit’ tab active, choose ‘Mask Selection’.
  b. Select the channel that needs the masking function. In our case, this is the channel for the Citrine staining which needs to be masked. Select ‘Mask Settings’>’Constant Inside/Outside’. After this, select ‘Set Voxels Outside Surface to’ and put 0.000 in the text box. This will create an additional channel, in which the intensity of the voxels that are outside the surface are set to zero.
  c. A new channel is created in order to inspect the masked channel, open the ‘Display Adjustment’ window with ‘Ctrl+D’ (or Edit>Show Display Adjustment), turn off other channels and select ‘Volume’ function.
    ***Note:*** The same procedure can be repeated for the TH channel. This step is not necessary for data analysis but helps to examine how well the mask was prepared and can help with data display. (Figure 8D)

40. Quantification of Citrine^+^ cells in the DA system. (See Methods video S1, 0:07:39)
  Spot detection provides a procedure to automatically detect and visualize point-like structures, and provide quantitative output of these structures. The Citrine^+^ cells in this ROI can be detected with spot analysis function.
  a. Start the ‘Spots’ wizard (3D View> Spots).
    (Icon shown in Figure 6)
  b. Perform spot analysis on the whole image. If the dataset is large, it is possible to select ‘Segment only a Region of Interest’ and followed by ‘Process entire image finally’.
  c. At page 2 of the ‘Spots’ analysis wizard (blue arrow), select the non-masked channel for Citrine staining.
    ***Note:*** When the analysis is performed in the masked channel, there is a sharp contrast between voxels within and outside the mask creating many false positive spots at the edges of the mask. For this reason, a non-masked Citrine channel was used for spot detection.
  d. Cell detection is facilitated by estimating the spot diameter in ‘Slice view’ (Ctrl+1) by measuring the diameter of a couple of cells by drawing a line between 2 extremes of a neuron cell body. Then enter the average value in ‘estimated XY diameter’. In our analysis the diameter used was 6 μm.
    ***Note:*** Since objects smaller than the estimated diameter are not detected as spots it is better to under- than overestimate cell size. For spot estimation, size variation in the desired cell population should not be too high, otherwise underestimation of cell diameter can result in multiple spots generated within a single large cell thereby skewing data analysis.
  e. Deselect ‘Model PSF-elongation along the Z-axis’. The Gaussian shape of the light sheet beam renders it nearly impossible to calculate point spread. Therefore, we choose to not model PSF in this analysis.
  f. Select ‘Background Subtraction’ to smoothen the image by creating a Gaussian filtered channel minus the intensity of the original channel.
    ***Note:*** For spot detection, a Gaussian filtered copy of the selected channel is created which uses a ratio of ¾ of the radius of the entered ‘Estimated Diameter’, to smoothen the image. When background subtraction is enabled a second Gaussian filtered copy of the original channel is made which uses a ratio of 8/9 of the radius of the entered ‘Estimated Diameter’. The second Gaussian blurred copy (8/9) is subtracted from the first blurred copy (3/4). This works as a difference of gaussians (DoG) algorithm and is useful for enhancing edges in the image and the suppression of noise.
  g. Page 3 of the spot analysis wizard (blue arrow) provides options to filter the spots. By default, the quality filter is selected. In addition to the quality filter (>300), we also added an intensity max (> 1500 on masked channel) and mean filter (>1000 on masked channel).
    ***Note:*** When background subtraction is disabled, the quality filter algorithm uses the intensity of the ¾ radius Gaussian filter channel (as described above). When background subtraction is enabled, it uses the intensity of the 2 subtracted gaussian filtered channels (DoG algorithm). The intensity is taken for the central voxels at a radius of 8/9 of the ‘Estimated Diameter’. Since the goal is to detect Citrine^+^ cells within the mask, we increased the stringency of spot detection by introducing two additional parameters for error-free spot detection. The intensity max selects cells with a certain brightness and intensity mean ensuring that the brightness value is spread over a defined cell area and is not a false positive pixel.
  h. Click on the green arrow to execute all the creation steps and to terminate the wizard. (Figure 8E)
    **CRITICAL:** Place the reference frame before performing spot detection. (See Troubleshooting section).

41. Spot segmentation along reference frame and analysis of Citrine^+^ subset within the DA system. (See Methods video S1, 0:10:14)
  This step provides information about the relative position of spots with respect to the reference frame. The reference frame rightly placed along x-y-z axes can provide data on the distribution of Citrine^+^ cells along the medio-lateral, rostro-caudal and dorso-ventral axis within the ROI (SN and VTA).
  a. The positional distance from the reference frame can be derived using the ‘Statistics’ tab in the spots menu, by selecting ‘Position Reference Frame’ from the dropdown menu (Statistics>Detailed>Position Reference Frame).
  b. Select ‘Export Statistics on Tab Display to File’. Export as *.xls* or *.csv.*
    (Icon shown in Figure 6)
  c. In the spreadsheet you will find the distance from the reference frame in all axes divided over three columns. These data can be used to make subgroups based on their distance from the reference frame.
    To be able to make subgroups based on their relative position from the reference frame it is necessary to determine which point along the axes of interest should be considered the end point. Measure the length of this axis by manually placing spots on the most lateral, rostral or ventral position of the surface.
  d. To measure the medial-lateral axis, open a new spots object (3D View> Spots).
  e. Then select ‘Skip automatic creation, edit manually’.
  f. Go to the ‘edit’ tab and select ‘Surface of Object’. This ensures that the spot is nicely placed on the outside of the surface.
  g. Press ‘Shift+left click’ to place the spot.
  h. From the ‘Statistics’ tab > ‘Selection’ tab, choose ‘Position Reference Frame’ from the dropdown menu. Retrieve the value for the specific axis you want to measure.
  i. The retrieved value is then used to subdivide the cells (spots) in groups/bins, to determine exact number of Citrine^+^ cells in each bin.
  j. Repeat this procedure for the other axes.

42. Graphical display of segmented groups within the DA system (optional). (See Methods video S1, 0:12:41)
  If a graphical display of the subgroups is desired, Imaris™ can be used to color-code each group using the following steps.
  a. Use the ‘Filter’ tab within spots to create new groups.
  b. Then add filter ‘Position X Reference frame’ or ‘Position Y Reference frame’ or ‘Position Z Reference frame’, to create medio-lateral, dorso-ventral and rostro-caudal subgroups, respectively.
  c. Determine the distances from the reference frame for each bin. For instance, for medial-lateral subgroups we used the most lateral position of the surface and the midline (medial position defined by the reference frame) and divided this region into 8 bins.
  d. In the upper threshold text box, enter the value of the farthest distance from the reference frame. In the lower threshold text box, enter the value of the nearest distance from the reference frame and click on ‘Duplicate Selection to new Spots’.
  e. Continue this action until there is a new subset for all bins created.
  f. For the sake of clarity all these groups can be put in one folder (3D View > Group). After the folder is created the spot subgroups can be manually dragged into this folder.
  g. Each subgroup can now be color-coded by a unique color by clicking on ‘Color’ tab, selecting ‘base’ and a unique color. (Figure 8F)
  h. Repeat this procedure for the dorso-ventral (Figure 8G) and rostro-caudal axis (Figure 8H).
    ***Note:*** Since version 9.6 of Imaris™ (July 2020) the division into groups can also be done at once, by selecting the classify spots function in the spot wizard. But the method described above can also still be used.

## Expected outcomes

Figure 2 shows an optimally cleared brain sample in comparison to a non-cleared sample (P0 mouse brain).

If the brain sample is cleared well (Figure 2B), stained and imaged, one should see clear staining when opening the file with a 3D viewer, as shown in Figure 8A (and Methods video S1). Here, DA neurons of the ventral midbrain (consisting of the brain areas VTA and SN) are clearly marked by TH staining and Citrine+ neurons are clearly visible so that they can be detected as individual neurons. TH staining should be clear enough to create a surface around the VTA/SN by manually drawing the surface (Figures 8B and 8C) so that the Citrine+ neurons within the VTA/SN areas can be masked (Figure 8D). After this individual Citrine+ neurons can be detected within the VTA/SN (Figure 8E) and these regions can be divided into bins based on their relative position from a reference frame (Figures 8F–8H).

At postnatal stages the surface drawn for the VTA/SN should be comparable to that shown in Figure 9A and should reflect the VTA/SN marked by the TH staining. It is therefore important to draw a sufficient number of planes (Figure 9B and 9C) and to not to skip too many planes, as shown in Figures 9E and 9F. This will cause the surface to not completely cover the VTA/SN (Figure 9D).

When the reference frame is placed with the aid of clearly recognizable histological landmarks, it should be possible to generate medio-lateral (Figure 8F), dorso-ventral (Figure 8G) and rostro-caudal (Figure 8H) bins for the Citrine+ neurons within the VTA/SN, which clearly reflect these directions (Figures 10C–10H and Methods video S1)

The information obtained about the position of Citrine+ neurons, relative to the reference frame, can ultimately be used to display the data in a graph, in which data from multiple samples can be combined. (see Brignani et al., 2020).

## Limitations

Migration analysis of specific neuronal subsets using this method can be performed only if the labeling of the neuron subtype of interest is sparse enough to prevent cells from being too tightly packed, which hampers identification of individual cells. Another limitation is that the number of fluorophore channels available for imaging is restricted due to tissue autofluorescence.

The whole mount immunostaining procedure is limited by the availability of primary antibodies that can fully penetrate brain tissue and produce specific staining patterns with low background staining. For the analysis of structures positioned in deep brain areas, we suggest to always perform a trial experiment trying different primary antibodies concentrations or even primary antibodies (including nanobodies, nanobodies generally penetrate tissue more easily due to their smaller size).

Note that the clearing procedure causes shrinkage of the sample. When several samples need to be compared (e.g., wild type and knockout brains), all samples must be cleared in parallel at the same time, to avoid differential shrinkage between samples.

Finally, the software can be unstable. When working with Imaris™ files, usage of small file sizes (by 3D cropping), converting files to 8-bit where possible, regularly saving “scenes” to facilitate easy save and reloading of data for analysis are all advisable methods for reducing crash incidence.

## Troubleshooting

### Problem 1

Samples look opaque or have a dark amber-like appearance after clearing (step 15). Possible reasons are 1) Blood retained in tissue, 2) Incomplete dehydration, 3) Oxidation of THF or DCM solution, and 4) Plastic incompatibility.

### Potential solution

Solution 1: For late embryonic and postnatal samples, PBS and PFA perfusion may help to remove the blood from vessels.

Solution 2: Use dehydration solutions that are freshly prepared.

Solution 3: Make sure that the THF is supplemented with BHT as an inhibitor and DCM with amylene as a stabilizer. The inhibitor and stabilizer prevent accumulation of peroxide byproduct accumulation which affects clearing. During THF and DCM treatment the tissue should be processed in a container filled with a minimum of air to prevent oxidation.

Solution 4: Use recommended glass/plasticware (like polypropylene (PP)). Polystyrene (PS) leaching in the sample will prevent clearing.

### Problem 2

Strong staining only at tissue edges (e.g., only in the skin) (step 36). Possible reason; Improper antibody penetration.

### Potential solution

Removal of a portion of the skin or meninges can improve antibody penetration.

### Problem 3

Appearance of bright ring (step 36). Possible reason; Suboptimal antibody concentration.

### Potential solution

Try to decrease antibody concentration. Very high antibody concentrations can cause poor diffusion.

### Problem 4

Bright blobs of staining in the tissue in a random pattern (step 36). Possible reason; Deposition of secondary antibody conglomerates in the brain tissue.

### Potential solution

Centrifuge the secondary antibody stock/antibody solution. Before use, filter the secondary antibody diluted solution using a 0.2 μm filter.

### Problem 5

No staining in deep brain regions, but good staining at the edges of the tissue without ring formation (step 36). Possible reasons are 1) Concentration of antibody is insufficient (most likely the primary antibody), and 2) Incubation time of the antibody (primary and/ or secondary) was too short.

### Potential solution

Solution 1: Consider increasing primary antibody concentration or trimming of ROI.

Solution 2: Consider increasing incubation time with primary and/or secondary antibody.

### Problem 6

Very high background, particularly in vasculature (step 36). Possible reasons are 1) Non-specific binding of antibodies, and 2) Blood retained in tissue as a consequence of incomplete or no perfusion of the tissue.

### Potential solution

Solution 1: Mouse antibodies often cause this issue, as anti-mouse secondary antibodies bind vasculature. For the application of new primary antibodies, we recommend to test primary antibodies from different vendors (For example, anti-TH antibodies raised in sheep from Pel-Freeze give better results in 3DISCO as compared to Millipore antibodies. Both antibodies work equally well in sections).

Solution 2: For late embryonic and postnatal samples, PBS and PFA perfusion may help to remove blood from vessels and to improve fixation.

### Problem 7

Weak or very light fluorescent signal (step 36). Possible reasons are 1) Inefficient antibody labeling, 2) Peroxide accumulation in clearing solution, 3) Low laser power/short exposure time, and 4) Sample was not well protected from the light after the secondary antibody (or conjugated primary) was added.

### Potential solution

Solution 1: Not all primary antibodies are compatible with tissue clearing and with long incubation at 37°C. Trials with multiple primary antibodies from different vendors is a useful investment. You could also experiment with incubations at lower temperatures.

Solution 2: Peroxide contamination is best prevented by using small stock volumes reducing the chance of contamination or removal of peroxide. Peroxide concentration can be checked using strips (see key resources table for details).

Solution 3: Increase laser power or exposure time.

Solution 4: Protect sample better from the light. It is recommended to use amber colored tubes, instead of wrapping your tubes in aluminum foil, since aluminum foil tears easily.

### Problem 8

Sudden shift/movement of the sample (step 36). Possible reasons are 1) The sample has moved during scanning (Most often this occurs when moving from one channel to the next), and 2) When the lasers do not derive from the same beam combiner, the input from the lasers may be misaligned (In our setup the 730 nm laser is in a separate beam combiner). In this case, images in one channel have a different starting point in Z than in the other channels, creating a shift in the Z direction.

### Potential solution

Solution 1: Tighten sample on the sample holder and reimage the sample (There is a tight balance between screwing your sample too loose or putting it too tight). When your sample drops from the holder into the cuvette then switch off the laser and ask your facility manager for help. Be very careful when taking the cuvette in/out of the microscope. DBE spillage can lead to destruction of the step motor of the light sheet. If your sample is small and not too irregularly shaped you can also embed your sample in agarose to facilitate mounting. (Be aware that mounting in agarose can also cause tissue damage).

Solution 2: When this occurs with a laser in the near infrared/ infrared spectrum, contact the vendor for support. The calibration tool does not support these wavelengths and special tools (prisms) are needed.

### Problem 9

Non-sharp image (step 36). Possible reasons are 1) Imaging out-of-focus, 2) Incorrect clearing solution selection in software. The position of the sheet focus depends on the refractive index of the liquid, and 3) The cuvette is not properly filled. A low level of clearing solution in the cuvette prevents the acquisition of a sharp image because the objective cannot be dipped into the clearing solution and the refraction is too high.

### Potential solution

Solution 1: Zoom digitally during imaging setup, put the horizontal focus on a thin feature (like an axon) and adjust the focus.

Solution 2: Select the correct imaging liquid (DBE, water etc.)

Solution 3: Switch off the laser and ask your facility manager for help to remove the cuvette from the microscope. Never fill the cuvette while it is located in the microscope chamber. Be very careful when taking the cuvette out of the microscope and when putting it in. DBE spillage can lead to the destruction of the step motor of the light sheet. Fill the cuvette till the max indication.

### Problem 10

Quick bleaching of fluorophores (step 36). Possible reasons are 1) High laser power for imaging, and 2) Re-imaging in multiple planes.

### Potential solution

Solution 1: For determining imaging setup it is recommended to increase exposure time instead of laser power to prevent bleaching. If the sample labeling is weak, a small increase of the laser power can cause bleaching. Be aware that laser intensity of the sheet is not only affected by laser power, but also by sheet width and NA.

Solution 2: Choose the best sample orientation before imaging, and avoid re-imaging in multiple planes. Take the following into consideration: 1) In general XY resolution is better than XZ and YZ resolution (if good resolution is needed in the sagittal orientation, then mount the sample in the sagittal orientation), 2) The longer excitation and/or emission light has to travel the more refraction will be caused, and 3) The working distance of the objective/dipping cap should be long enough to image the full ROI.

### Problem 11

Tissue damage (step 1, 23, 36). Possible reasons are 1) Tissue was damaged during tissue dissection. Tissue damage is increased by dehydration and clearing, and 2) Tissue was damaged during mounting.

### Potential solution

Solution 1: Be careful during sample dissection. Perform the isolation of embryonic brains with a dissection microscope, while keeping the tissue at 4°C.

Solution 2: Try not to screw your sample too tight into the holder. After clearing, the tissue gets glass-like properties and it easily breaks. It sometimes helps to support the tissue with small cleared agarose blocks (By cutting agarose on a vibratome to slices of 0.5–1 mm you can create evenly proportioned blocks of agarose). By sanding the tip of the screw, you can make it more blunt preventing damage to the tissue.

### Problem 12

Air bubble (step 23). Possible reasons are 1) When lowering your sample or the objective into the clearing solution (DBE) an air bubble was accidently introduced in the DBE fillet imaging cuvette, and 2) An air bubble formed in the sample during tissue clearing.

### Potential solution

By moving your sample with the microscope joystick you can see if this air bubble is in the clearing solution or in the sample itself. When the air bubble moves in the same direction as your sample, the air bubble is most likely localized in the sample.

Solution 1: If the air bubble does not disappear by itself, then move the objective out of the cuvette and check if the air bubble has disappeared. Otherwise try to remove the air bubble with forceps.

Solution 2: Take the sample out of the microscope and try to remove the air bubble by moving the sample. If the air bubble cannot be removed, try to image the sample with the least disturbance of the ROI (The air bubble will lead to stripes opposite of the origin of the laser). Check the following: i) If the ROI is on one side of the sample, use unidirectional sheet illumination and select illumination direction at the side of your ROI. ii) If the entire sample needs to be imaged, use bidirectional sheet illumination, and check whether the following options can minimize the (stripe) effect of the air bubble: 1) Try to put the region where the images of the right and left sheet illumination are blended around the air bubble, or 2) Use the contrast mode, instead of the default blend mode, to combine the images of the right and left sheet illumination, where the image from one side could compensate the image with the shadow effect. *Be aware that changes in illumination or algorithm can affect the analysis.*

### Problem 13

Cells are elongated along the Z direction (step 36). Possible reasons are 1) The magnification selected in the software was different from the magnification used for imaging. This causes incorrect positioning of the light sheet beam waist when using dynamic horizontal focus, because the position of the sheet is dictated by the (effective) magnification, 2) An incorrect instrument mode was selected. In the instrument mode the objective and/or type of dipping cap can be selected. This contributes to the (effective) magnification (In our setup the difference in magnification between a correction dipping cap and standard dipping cap is 7%). This also leads to an incorrect positioning of the light sheet beam waist when using dynamic horizontal focus, 3) Sheets are misaligned, and 4) Sheet motor calibration is improper or broken.

### Potential solution

Solution 1: Select the correct magnification in the software.

Solution 2: Select the instrument mode which corresponds to the dipping cap and/or objective.

Solution 3: Ask your facility manager to check the alignment of the lateral and middle sheets with the alignment tool.

Solution 4: Ask your facility manager to check with the alignment tool if the sheet motor calibration is correct, if not, change this in the software and save settings. In case the sheet motor is broken contact the vendor.

### Problem 14

Only one side of the image is in focus, when using bidirectional sheet illumination (step 23). Possible reason; Left and right sheets are not well aligned.

### Potential solution

Ask your facility manager to check the alignment of the left and right sheets with the alignment tool. (When using bidirectional sheet illumination, always check if a proper left/ right image can be acquired before starting the scan.)

### Problem 15

Shadows in the image (step 23). Possible reasons are 1) Air bubble in your sample (See section problem 12), 2) Pigmentation, and 3) Blood hematoma(s).

### Potential solution

Solution 1: See section problem 12

Solution 2: Try to improve image quality by following the suggestions described in at Problem 12. When starting new stainings, ensure that the sample is bleached well with H_2_0_2_.

Solution 3: See solution above. When starting new stainings, perform perfusion of the pups/ embryos and confirm proper bleaching with H_2_0_2_.

### Problem 16

The intensity of the signal is weaker at the bottom (step 23). Possible reasons are 1) The sample is not cleared well, and 2) The Z range of the sample is too high. The longer the distance excitation light has to travel through the tissue the more refraction there is. In addition, with shorter wavelengths this issue is worse.

### Potential solution

Solution 1: Try to put the ROI close to the objective to reduce refraction of the emission light by the tissue (See Figures 3C and 3D). Try to minimize the Z range by trimming the sample with a razor blade. Further it is possible to setup a power adaption along Z.

Be aware that changes in illumination can affect the analysis.

Solution 2: See solution 1.

### Problem 17

Sample only has a good Z resolution in the middle of the sample (step 36). Possible reasons are 1) Dynamic horizontal focus is off, 2) Number of dynamic horizontal focus steps was set to 1, and 3) The range for dynamic horizontal focus was set only to the middle part of the image.

### Potential solution

Solution 1: Switch on dynamic horizontal focus.

Solution 2: Increase the number of horizontal focus steps.

Solution 3: Set the range for dynamic horizontal focus so that it covers the entire ROI.

### Problem 18

Vertical stripes (which coincide with the selected number of dynamic horizontal focus steps) (step 36). Possible reasons are 1) The number of horizontal focus steps was too low to match the sheet NA, 2) Calibration of the sheet motor is incorrect or the sheet motor is broken, 3) The magnification selected in the software was different than the magnification used for imaging. This causes incorrect positioning of the light sheet beam waist when using dynamic horizontal focus, because the position of the sheet is dictated by the (effective) magnification, and 4) The incorrect instrument mode was selected. In the instrument mode the objective and/or type of dipping cap can be selected. This contributes to the (effective) magnification (In our setup the difference in magnification between a correction dipping cap and standard dipping cap is 7%). As in 3. this also leads to incorrect positioning of the light sheet beam waist when using dynamic horizontal focus.

### Potential solution

Solution 1: Either increase the number of horizontal focus steps or decrease sheet NA (which leads to a broader beam waist but while the beam waist is stretched over a longer range)

Solution 2: Check the alignment of the sheet motor with the alignment tool, if this appears to be incorrect adjust the sheet motor calibration within the Imspector software and save the settings. In case the sheet motor is broken contact the vendor.

Solution 3: Select the correct magnification in the software.

Solution 4: elect the instrument mode, which corresponds to the dipping cap and/or objective.

### Problem 19

Cells in the center are duplicated in the Z direction when using bidirectional sheet illumination (step 36). Possible reasons are 1) Left and right sheets are not aligned well, and 2) If other algorithms than blend are used to merge bidirectional illumination this can occur.

### Potential solution

Solution 1: Ask your facility manager to check the alignment of the left and right sheets. (When using bidirectional sheet illumination always check if you get a proper left/right image before you start your scan.)

Solution 2: Select the blend algorithm to merge bidirectional illumination.

### Problem 20

Strong illumination in the center along the X axis (step23, 36). Possible reason; Sheet width was set too low, therefore only the center along the x axis is strongly illuminated.

### Potential solution

Set sheet width such that the complete sample or ROI is evenly illuminated (If you are not already using maximum laser power it is better to overestimate sheet width a bit to keep the illumination as even as possible) (See Figure 5).

### Problem 21

Only one channel in focus (step 36). Possible reason; There is no chromatic correction performed for each channel or it was set incorrect. Only the channel in which you focused before imaging is now in focus.

### Potential solution

Focus in the channel with the lowest wavelength and hereafter change the chromatic correction in the software for the other channels.

### Problem 22

Samples look flat in Imaris™ (step 36). Possible reason; Meta-data with scaling information is missing in the Imaris™ file. Probably the Imaris™ file was created without the first file of the ome.tiff series (ending with C00_z0000.ome.tif). Only this tiff contains (∗depending on your auto save settings) the metadata for the entire scan.

### Potential solution

Recreate the Imaris™ file with the first image of the scan in Imaris™ file converter (ending with C00_z0000.ome.tif). Or add the missing scaling info via the Image Properties window (Ctrl + I).

### Problem 23

Incorrect scaling (step 36). Possible reasons are 1) The magnification selected in the software was different than the magnification used for imaging, this causes incorrect calculation of pixel size, and 2) Incorrect instrument mode was selected. In instrument mode the objective and/or type of dipping cap can be selected. This contributes to the (effective) magnification (In our setup the difference in magnification between a correction dipping cap and standard dipping cap is 7%). As in 1. this causes incorrect calculation of pixel size.

### Potential solution

Solution 1: Although it is possible to fill the imaging properties in Imaris™ (Ctrl+I), it is advisable to repeat the imaging and select the correct magnification in the microscope software. The imaging settings are likely affected by the wrong magnification input.

Solution 2: Although it is possible to fill the imaging properties in Imaris™ (Ctrl+I) it is advisable to repeat the imaging and select the instrument mode which corresponds to the dipping cap and/or objective. Incorrect instrument mode input likely affects the image settings.

### Problem 24

Reference frame not detected in spot statistics (step 41) . Possible reason; The reference frame was not added before spots were created.

### Potential solution

Select all spots and duplicate them. Now check statistics from the duplicated spots wizard.

### Problem 25

Reference frame doesn’t match its actual position in spot statistics (step 41). Possible reason; The reference frame was not set at its final location before spots were created.

### Potential solution

Select all spots and duplicate them. Now check statistics from the duplicated spots wizard.

### Problem 26

Different intensities among samples (step 40). Possible reason; Microscope settings differed between samples. The intensity is not only determined by laser power and exposure time, but also by sheet width and NA. (Be aware that when using dynamic horizontal focus the exposure time will be (automatically) adjusted to match the travel time of the focus in order to synchronize camera acquisition with movement of the light sheet focus).

### Potential solution

A new imaging session is required. Use the same imaging parameters for all samples.

## Resource availability

### Lead contact

Further information and requests for resources and reagents should be directed to and will be fulfilled by the lead contact, R. Jeroen Pasterkamp (R.J.Pasterkamp@umcutrecht.nl).

### Materials availability

*ACTB-Flpe:Pitx3-ITC* mice are available from the lead contact.

### Data and code availability

Raw data of imaged brain sample files can be procured from the lead contact upon request.
